# Validity of microscopy for diagnosing urinary tract infection in general practice – a systematic review

**DOI:** 10.1080/02813432.2019.1639935

**Published:** 2019-07-14

**Authors:** Anja Kofod Beyer, Gloria Cristina Cordoba Currea, Anne Holm

**Affiliations:** Research Unit for General practice and Department of General Practice, University of Copenhagen, København, Denmark

**Keywords:** Microscopy, general practice, outpatient, UTI, urine tract infection

## Abstract

**Objective:** To investigate the validity of microscopy as a diagnostic tool for urinary tract infection in general practice.

**Methods:** (Design/setting) A systematic review was conducted by searching Medline for clinical studies made in general practice, outpatient clinics or similar settings in which the accuracy/validity of microscopy was evaluated with urine culture as the reference standard.

**Results:** Our search resulted in 108 titles. 28 potentially eligible studies were retrieved for full-text reading. We included eight studies involving 4582 patients in this review. The quality of the studies was moderate to high. Specificity ranged from 27% to 100%, sensitivity from 47% to 97%. The variation between studies did not allow for meta-analysis.

**Conclusion:** We did not find substantial evidence to determine the clinical validity of microscopy performed in general practice on urine samples from patients with symptoms of UTI.

**Key points**
Urinary tract infection is common in general practice. Methods for precise diagnosis are needed in order to avoid inappropriate treatment.

- Currently no evidence-based consensus exists regarding the use of urinary microscopy in general practice.
- We did not find substantial evidence to determine the overall clinical validity of microscopy performed in general practice on urine samples from patients with symptoms of UTI.
- Light microscopy with oil immersion had high sensitivity and specificity but is time-consuming. Phase-contrast microscopy is quick and had high specificity but lower sensitivity.

## Introduction

Urinary tract infection (UTI) is common in general practice [1]. UTI symptoms often results in prescriptions of antibiotics [2]. Most antibiotics are prescribed in the primary health care sector and consumption is associated with the development of antibiotic resistance [3]. In order to properly manage UTIs in general practice, quick, precise and low-cost diagnostic tools are needed.

Diagnosing UTI using clinical history and urine dipstick is a common strategy in most countries, but these approaches result in a high rate of false positives leading to overtreatment with antibiotics [4,5]. The reference standard for diagnosing UTI is urine culture [6]. However, this modality is more time consuming and while waiting for the result treatment is often initiated empirically [7].

A rapid and accurate test for identification of patients at high or low risk of having bacteria in the urine is needed. Point of care (POC) microscopy is an alternative rapid-test for this purpose, which is frequently used in Danish general practice [8].

POC microscopy using either a traditional light microscope or a phase-contrast microscope enables the clinician to quantify the number of bacteria seen per field of vision, determine the morphology of the organisms (rod or cocci), and describe their type of motility (non-motility, polar or non-polar motility) [9,10]. Phase-contrast microscopy has the advantage of not requiring staining of the specimen. Several studies have investigated the validity of POC microscopy [8,9], and the accuracy of microscopy in the laboratory setting has been investigated[10], but the clinical validity of POC microscopy in general practice has not previously been summarized in a systematic review.

The aim of this systematic review was to determine the clinical validity, i.e. sensitivity and specificity, of microscopy performed in general practice on urine samples from patients with symptoms of UTI, using urine culture as a reference standard.

## Method

### Literature search

A systematic search of the literature was performed using the bibliographic database Medline. Medline was searched for scientific articles in English, Swedish, Norwegian and Danish, restricting the search to studies published in 1975 or later. Search words included urinary tract infections, cystitis, bacteriuria, microscopy, primary health care, general practice, family practice and outpatient clinic. The full search string can be seen in Appendix A. The literature search and inclusion of studies was performed by two independent authors (AB and AH), in August 2017. Additionally, AH and GC searched the reference lists of the included articles, to detect reports of studies not found in the database search, and AH asked one knowledgeable expert to identify any additional studies. When data was not available or incomplete, we refrained from contacting authors, as most studies were more than 10 years old.

### Inclusion criteria

Diagnostic studies, in which the accuracy/validity of urine microscopy on urine from patients with symptoms of urinary tract infections performed in general practice, outpatient clinics or a similar setting by the GP or general practice staff with urine culture at the microbiological department as reference standard were eligible for inclusion.

### Data extraction

Information on publication date, setting, sample size, country, inclusion criteria, type of urine sample, staining, type and power of microscope, sediment, number of fields examined, how the study defines infection in both microscope and reference standard was extracted independently by AB and AH, and discrepancies were discussed and corrected. If these measures were not directly provided in the article, we calculated them if possible. Data on absolute numbers of true and false positives and negatives or predictive values on urine microscopy was extracted. When only one was reported, the others were calculated. To allow dichotomization, culture results presented as equivocal and contaminated were grouped with the negative results, because these three results usually have the same clinical consequences.

### Quality assessment

The risk of bias of included studies was assessed by using the QUADAS-2 tool [11]. Quality assessment was done independently by GC and AB, and discrepancies were discussed and corrected. Studies with moderate quality according to QUADAS-2 were not excluded from the review.

## Results

### Literature search

**Figure 1.**
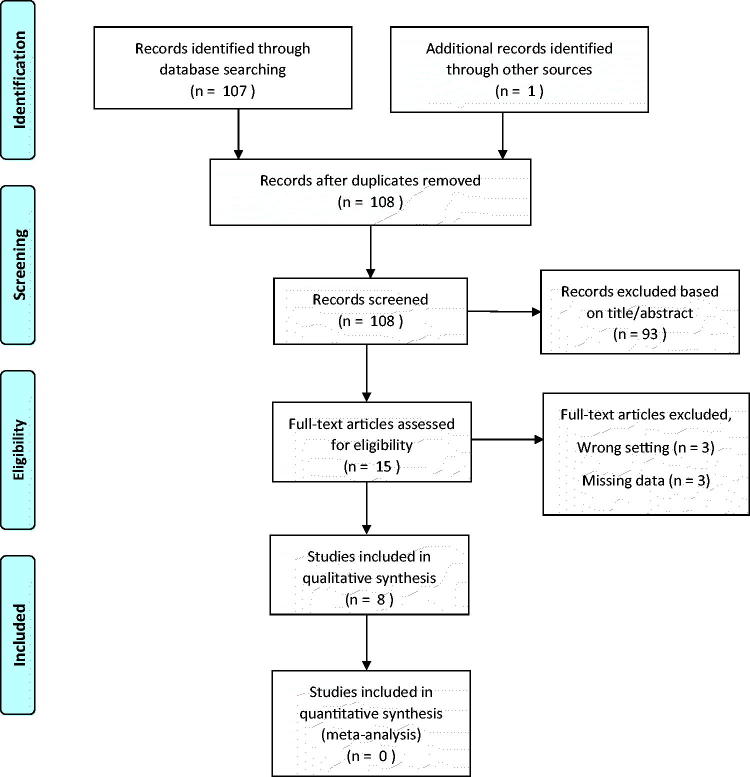
Flow diagram illustrating selection of studies.

108 potentially eligible studies were identified searching Medline. After full text reading 100 studies were excluded thus resulting in eight eligible studies (Figure 1). The main reasons for not including studies were studies using a different design than described in the inclusion criteria and studies conducted in the secondary sector. The included studies were performed in the period between 1979 and 2015 and involved 4582 patients (from 100 to 1663 patients per study).

### Quality of included studies

Four of the studies were judged to have moderate risk of bias. Four studies were considered having low risk of bias. The most common error was in the process of patient selection (not a consecutive sample). The quality of the included studies is summarized in Table 1.

**Table 1.** Quality of included studies.

Study	Risk of bias				Applicability concerns		
	Patient selection	Index test	Reference standard	Flow and timing	Patient selection	Index test	Reference standard
Chalmers [12]							
Winkens [13]							
Ditchburn [14]							
Ferry [15]							
Hallander [16]							
Balslev [17]							
Dornfest [18]							
Wilks [19]							

### Validity of POC microscopy

The studies differed in which technique of microscopy was used, in how many practices were included, and in what cut-offs they used for measuring infection. Table 2 shows characteristics of the identified studies and Table 3 shows the validity of the investigated techniques. The studies used different methods for urine sampling. Midstream clean-catch (MSCC) was used by two of the studies, midstream urine (MSU) was used by three of the studies and the rest did not specify which method they used for urine sampling. Five studies used light microscopy, one study used phase-contrast microscopy and two studies did not specify which type of microscopy they used.

**Table 2.** Characteristics of included studies.

Study	Year	Country	Patients(n)	Setting	Urine sampling method	Type of microscopy	Magnification	Staining	Sediment	Measure of infection microscopy	Measure of infection culture
Dornfest [18]	1979	South Africa	109	Single practice	MSCC^a^	Light microscopy	×1000	Yes	Yes	≥35 organisms in total over 5 fields	>10^e^ bacteria per ml
Wilks [19]	1979	Great Britain	100	Single practice	MSCC^a^	Light microscopy	×110	No	No	≥1 white blood cell per LPF^b^	>10^e^ bacteria pr ml
							×490			≥1 motile bacillus per HPF^c^	>10^e^ bacteria pr ml
Ditchburn [14]	1990	Great Britain	237	Single practice	MSU^d^	Light microscopy	×75	No	No	≥18 leukocytes per LPF^b^	>10^e^ bacteria pr ml
Balslev [17]	1980	Denmark	1663	Multi practice	Any^e^	NA^f^	NA^f^	NA^f^	Yes	Pyuria or bacteria^g^	>10^e^ bacteria pr ml
Hallander [16]	1986	Sweden	776	Single practice	Any^e^	Phase-contrast microscopy	NA^f^	NA^f^	Yes	Moderate or abundant bacterial finding per field	>10^e^ bacteria pr ml
										≥20 white blood cells per field	>10^e^ bacteria pr ml
Winkens [13]	1995	Netherlands	1311	Multi practice	Any^e^	NA^f^	NA^f^	NA^f^	Yes	≥5 leukocytes	>10^e^ bacteria pr ml
										≥20 Bacteria	>10^e^ bacteria pr ml
Ferry [15]	1990	Sweden	201	Single practice	MSU	Light microscopy	×400	Yes	Yes	≥5 leukocytes per HPF or 100-300 bacteria per HPF	>10^e^ bacteria pr ml
Chalmers [12]	2015	Thailand	185	Single practice	MSU	Light microscopy	×40 + 10	Yes	Yes	≥10 white blood cells per HPF and ≥1 bacteria per HPF	Significant growth

a MSCC: Midstream clean-catch.

b LPF: Low power field.

c HPF: High power field.

d MSU: Midstream urine.

e Any: Without any specific urine sampling method.

f NA: Not avaliable.

g Not reporting any cut-offs.

**Table 3.** Accuracy summaries of included studies.

Study^a^	Prevalence (%)	PPV (%)	NPV (%)	SEN (%)	SPE (%)	LR +	LR −
Dornfest [18]	28	85	97	93,5	93,6	14,59	0,07
Wilks [19]	68	100	48	48,5	100		0,51
	33	55	88	81,8	67,2	2,49	0,27
Ditchburn [14]	41	74	95	94,9	76,3	4,00	0,07
Balslev [17]	48	75	85	85,7	73,7	3,261	0,194
Hallander [16]	17	87	95	74,0	97,0	24,67	0,27
	17	65	92	60,0	93,0	8,57	0,43
Winkens [13]	69	73	58	91,9	27,0	1,248	0,332
		85	41	47,0	81,0	2,476	0,655
Ferry [15]	82	88	74	97,0	38,9	1,59	0,08
Chalmers [12]	42	79	74	57,1	88,9	5,14	0,48

a Most of the studies did not have enough data available to calculate 95% confidence intervals.

The prevalence of UTI varied between 17–82% in the eight studies. Sensitivity (SEN) in the studies varied between 47% and 97%. The specificity (SPE) was varying between 27% and 100%. The positive predictive value (PPV) of microscopy was high in most of the studies, between 73% and 100%. The negative predictive value (NPV) varied between 41% and 97%.

The study using light microscopy with oil immersion gave the highest clinical accuracy (SEN 94%, SPE 94%) [18]. Phase-contrast microscopy had a high SPE (97%) but a lower SEN (74%) when the index test was interpreted as positive based only on bacteria, and an even lower clinical accuracy (SEN 60%, SPE 93%) when the index test was interpreted as positive based only on leukocytes [16]. A Danish study from 1980 on 1663 patients investigating any microscopic technique used at that time in Danish general practice showed similar results (SEN 74%, SPE 97)[17].

## Discussion

### Statement of principal findings

This systematic review did not find consistent evidence to determine the clinical validity of microscopy in general practice. Due to pronounced heterogeneity of the studies, they were difficult to compare. However, one study using oil immersion light microscopy showed high clinical accuracy. Another study involving 776 patients using phase-contrast microscopy, which is the technique most commonly used in Denmark today, showed high specificity but a low sensitivity. The quality of the studies was moderate to high.

### Strengths and weaknesses of the study

This systematic review is the first to systematically assess the literature on the accuracy of POC microscopy as a diagnostic tool for patients with symptoms of UTI in general practice. Two persons reviewed the literature on urine microscopy, attempting to include all available original studies dealing with microscopy in general practice. We only included studies performed in general practice or similar and not studies made in other settings, so the result is applicable to all general practices. In the included studies, patients had symptoms of UTI much like the typical UTI-patient in general practice. Thus, we believe the results can be considered relevant to all general practices. The review identified both single-center and multicenter studies.

The included studies varied in microscopic technique and applied cut-off, being both an advantage by adding diversity to the review and a disadvantage because of the difficulty in comparing the studies. Unfortunately, this variation did not allow us to perform meta-analysis. None of the studies used todays cut-off of 10^3^ cfu/ml for primary uropathogens in the reference test. The studies did not describe a screening process of the urine samples at the laboratory, but it is possible that the negative urine-microscopy sometimes was confirmed by another urine-microscopy at the lab in which case the study would be expected to yield falsely high accuracy measures. Also, significant growth in urine culture from a symptomatic patient can be questioned as a good reference standard for UTI. However, this is the most commonly used definition and it has some clinical value since patients without significant bacteriuria tend to recover faster without treatment than those with [20,21]. Since phase-contrast microscopy is only able to identify quantities of bacteria down to 10^5^ cfu/ml, this would be expected to affect sensitivity negatively. The risk of bias in the process of patient selection was high in some of the studies. Furthermore, the prevalence of UTI varies a lot between the studies (17% and 82%), this could be explained by inclusion of patients, whom may have had doubtful symptoms or been asymptomatic although the studies stated all patients were symptomatic. However, the variation in the prevalence of significant bacteriuria across countries and studies is consistent with findings in previous studies [22].

The number of studies on each technique did not allow us to make robust conclusions about the clinical validity of microscopy in general practice. However, to our best knowledge, the most common microscopy methods in general practice today in Scandinavia is either light microscopy of centrifuged, unstained urine with a magnification of x400 or phase-contrast microscopy of un-centrifuged, unstained urine with the same magnification. This review succeeded in finding literature on both methods.

### Findings in relation to other studies

Microscopy of urine for bacteria and leukocytes has been thoroughly investigated in the hospital setting both with and without centrifugation and staining and found that oil-immersion microscopy on centrifuged, gram-stained urine is most precise [10]. One study in this review used this method and did have the highest clinical accuracy. However, this procedure is time-consuming and may not be feasible in most general practices.

Evidence from the secondary sector is often used when implementing new diagnostics in general practice. The validity of the tests is often misjudged, because of the predictive values are influenced by the prevalence and the difference between patients and doctors in the two sectors [23,24]. However, the results in this review did not differ substantially from secondary-sector evidence since the validity of microscopy varied greatly in both sectors.

Dipstick is the only alternative for rapid on-site diagnostics for UTI in general practice. One large review on the validity of urine dipstick found dipstick testing to be useful as a screening test in general practice but lead to high levels of overtreatment if other diagnostics were not applied [25]. A large clinical study performed in general practice using todays cut-off of 10^3^ cfu/ml in the reference found about the same [5].

A systematic review from 2010 found that using urine microscopy as an add-on to urine dipstick slightly improved the sensitivity [6]. However, this combination still seems to be inferior to urine culture in order to avoid over-diagnosis [26].

Another POC testing method is POC culture performed in general practice. One study on POC dipslides have shown sensitivity and specificity of 88% and 90%, respectively [27]. Another study on dipslide found a lower sensitivity (73%) and specificity (94%) [28]. A study from 2017 on point-of-care culture using chromogenic agars in general practice found a sensitivity of 88% but a specificity of 55% [29]. Compared to urine dipstick, this method has the disadvantage of longer waiting time before getting the result of the culture.

New diagnostic POC testing methods, such as rapid immunoassay test, are being developed and could have a place in testing for UVI in future studies [30].

## Conclusions

This review did not find solid evidence to determine the clinical validity of microscopy performed in general practice on urine samples from patients with symptoms of UTI. The lack of evidence is due to few available studies, wide variation of the cut-offs for the index test, the level of magnification and the method of microscopy.

Immersion oil microscopy gave the highest clinical accuracy and phase-contrast microscopy had high specificity and could possibly be a good add-on test to urine dipstick to avoid over-diagnosis. With the current available evidence, phase-contrast microscopy seems to be a valid and feasible screening-test for bacteriuria in patients with symptoms of UTI in general practice. When general practices use urine microscopy, they should always send urine to culture when in doubt. Future studies on current methods for urine microscopy should use predefined cut-offs and algorithms to investigate if microscopy could be a cost-effective add-on test to urine dipstick in order to avoid both antibiotic overtreatment and unnecessary urine cultures.

## Appendix A

((“Urinary Tract Infections”[Mesh] OR “Cystitis”[Mesh] OR “Urinary Tract Infection” OR “Cystitis” OR “Bacteriuria”[Mesh] OR “Bacteriuria”) AND (“Microscopy”[Mesh] OR Microscopy OR microscope OR microscopic OR sediment) AND (“Primary Health Care”[Mesh] OR “Primary Health Care” OR “General Practice”[Mesh] OR “General Practice” OR “Family practice” OR “General practitioners”[Mesh] OR “general practitioner” OR outpatient*) NOT(“Animals”[Mesh] NOT “Humans”[Mesh]))
